# Supplementary material for: The dorsal blastopore lip is a source of signals inducing planar cell polarity in the Xenopus neural plate
Source: Biol Open. 2021 Jul 19;10(7):bio058761. doi: 10.1242/bio.058761 (PMC8325942; doi:10.1242/bio.058761)
Supplement: Supplementary information [file biolopen-10-058761-s1.pdf]

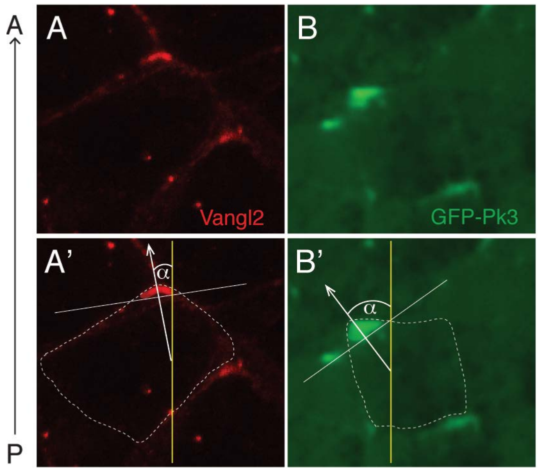

Figure S1. Scoring PCP aggregate orientation in the neural plate.

(A, B) Neuroepithelial cells exhibit anteriorly polarized endogenous Vangl2 (immunofluorescence) (A), or exogenous GFP-Pk3 (GFP fluorescence) in the presence of exogenous Vangl2 (unlabeled) (B). (A', B') Scoring method for the angle (α) that represents the orientation of the polarity vector (white arrow) for Vangl2 (A') and GFP-Pk3 (B') aggregates.

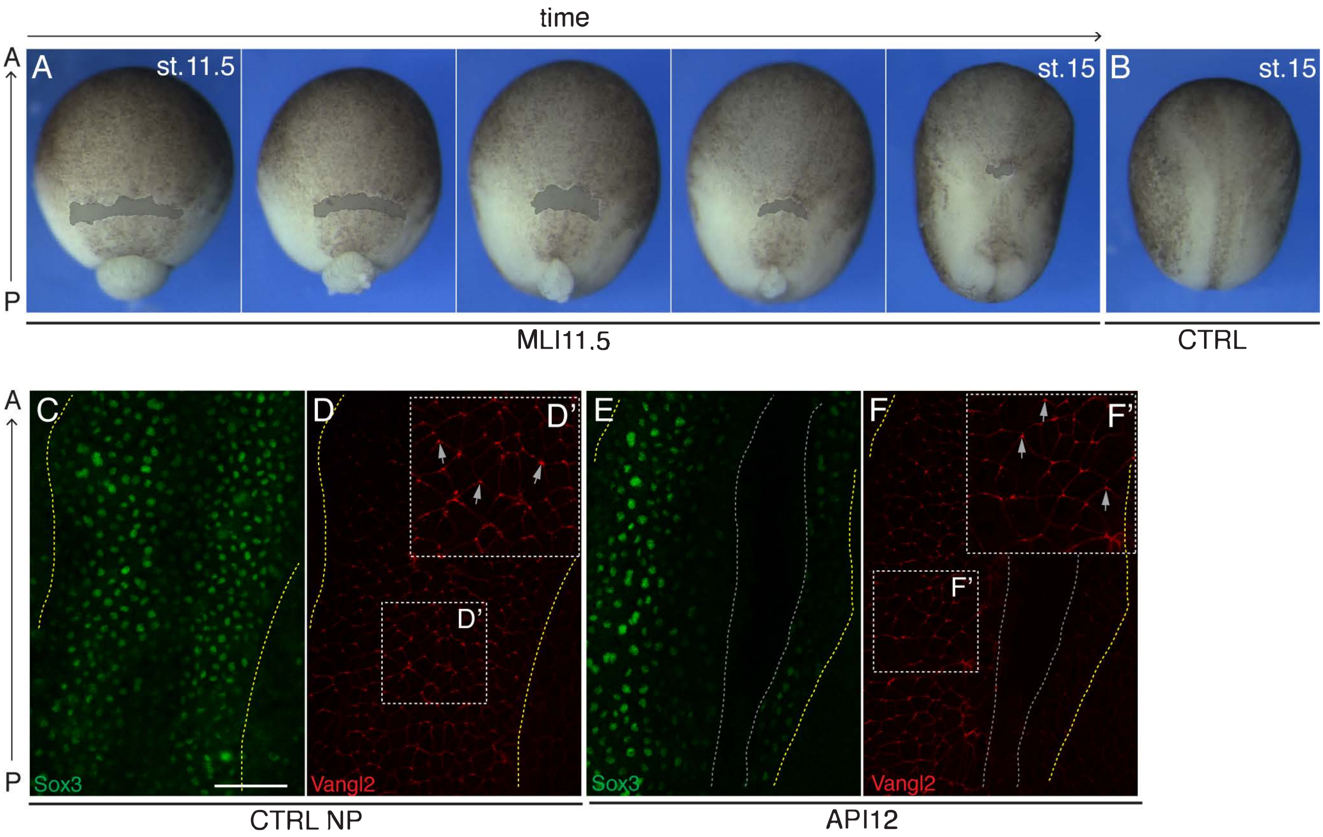

Figure S2. Embryo morphology and Vangl2 polarization after microsurgical incisions.

(A) Brightfield timecourse images of an embryo (stages 11.5-15) with mediolateral incision (MLI) made at stage 11.5. Note the changes in embryo and wound shape. (B) Control embryo at stage 15.

(C-F') Sox3 and Vangl2 immunostaining of stage 15 control embryo (C, D) and embryo with anteroposterior incision (API12) (E, F) made at stage 11.5/12. Boxed regions in (D, F) are magnified in (D', F'). Yellow lines delimit the neural plate, grey arrows show Vangl2 crescent orientation. Grey lines in (E, F) indicate the incision. The data are representative of three independent experiments with 3-6 embryos per group.

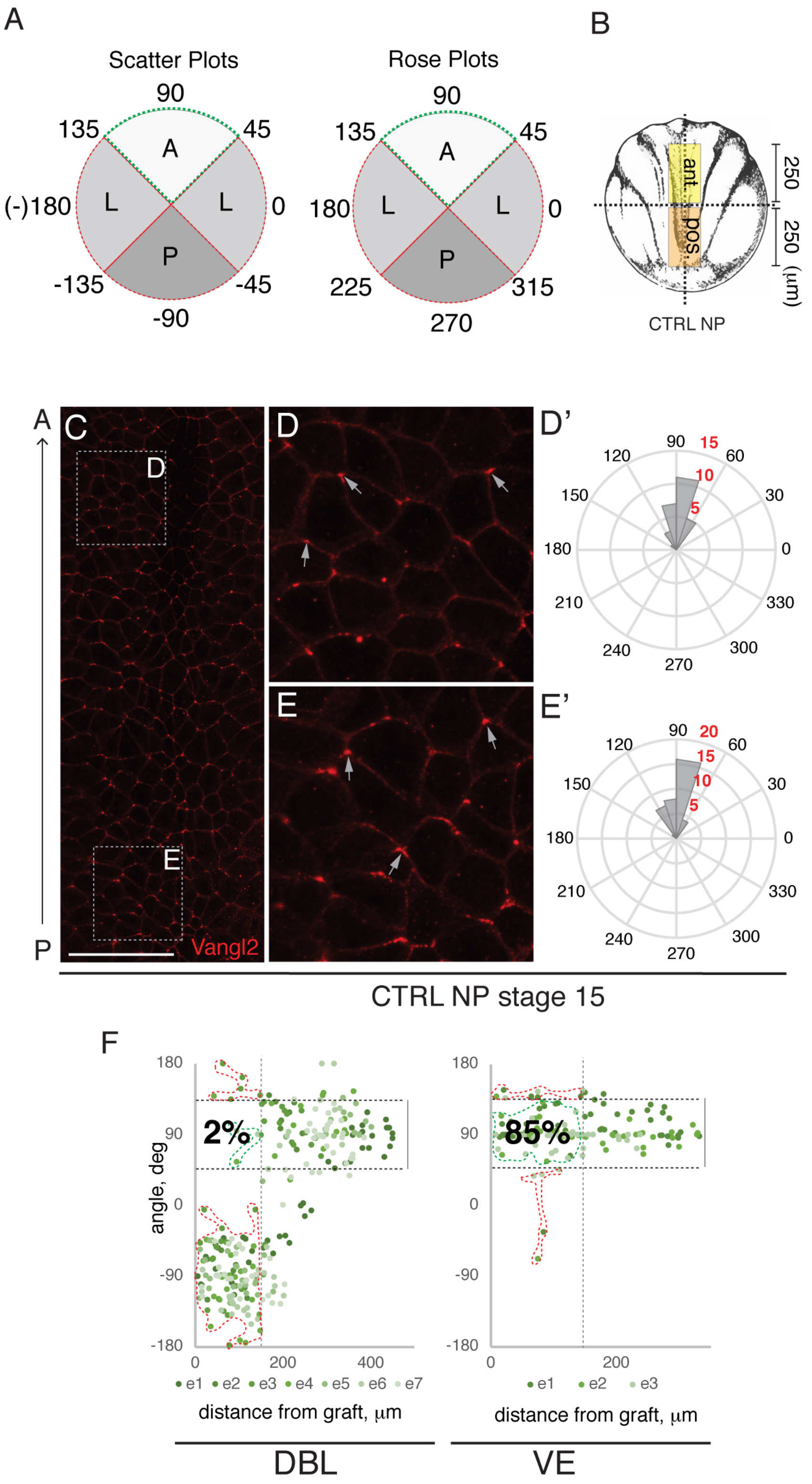
A
Scatter Plots
90
135
45
A
(-)180
L
L
0
P
-135
-45
-90
Rose Plots
90
135
45
A
180
L
L
0
P
225
315
270
B
ant.
pos.
250
250
(µm)
CTRL NP
A
P
C
D
E
Vangl2
D
D'
E
E'
CTRL NP stage 15
F
2%
85%
angle, deg
distance from graft, µm
e1 e2 e3 e4 e5 e6 e7
e1 e2 e3
DBL
VE

Figure S3. Quantification of Vangl2 aggregate orientation in the neural plate.

(A) Schematic representation of the methods used: scatter plots and rose plots (see Methods for detailed description). (B) Scheme of the neural plate with indicated areas of scored Vangl2 orientation. (C-E') Quantitation of Vangl2 polarization in the neural plate (NP) at stage 15. (C) Vangl2 immunostaining of a control NP at low magnification. Boxed areas in correspond to the anterior and posterior regions magnified in D and E, respectively. Grey arrows indicate Vangl2 crescent orientation. Scale bar in C, 100 μm. (D', E') Rose plots showing Vangl2 aggregate orientation. (F) Scatter plots of Vangl2 polarization in embryos grafted with DBL and VE. PCP vector is shown as function of the distance from the transplant. Shades of green represent different embryos. Green dots between black dashed lines represent cells with anteriorly localized Vangl2 crescents. Dashed shapes indicate normal (green) or altered (red) orientation. Grey dashed vertical line marks 150 μm from the graft. Percentages refer to the number of cells with normal anterior orientation within 150 μm from the graft.

Biology Open • Supplementary information

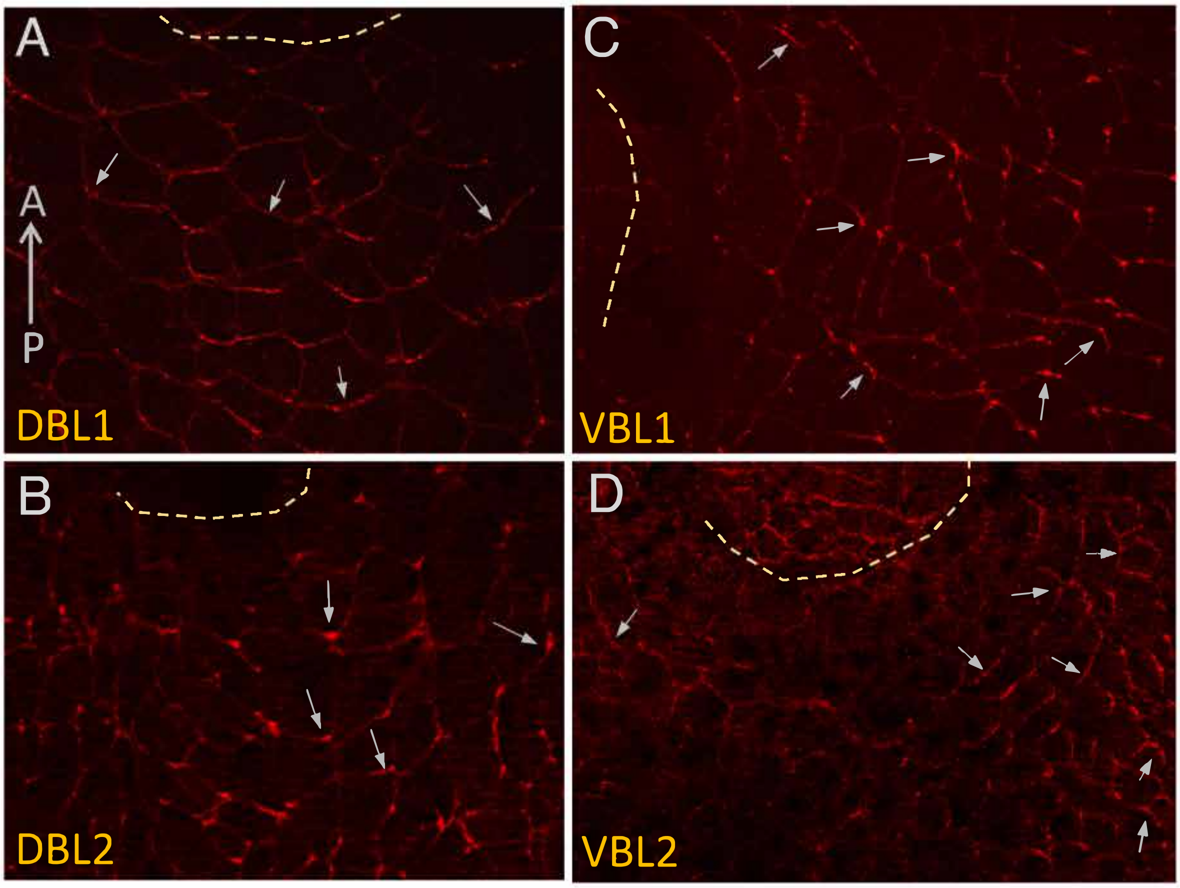

Figure S4. Both dorsal and ventral blastopore lip grafts cause PCP reversals in recipient neural plates.

(A-D) Vangl2 crescent orientation in the proximity of DBL (A, B) and VBL (C, D) grafts. Grafting was done as described in Figure 4. Two NP immunostained for Vangl2 are shown for each type of graft, which are representative of 4-7 independent experiments. Grey arrows indicate individual cell polarity based on Vangl accumulation. Approximate graft positions (dashed lines) and the orientation of the anteroposterior (AP) axis (refers to all panels) are shown.

Biology Open • Supplementary information

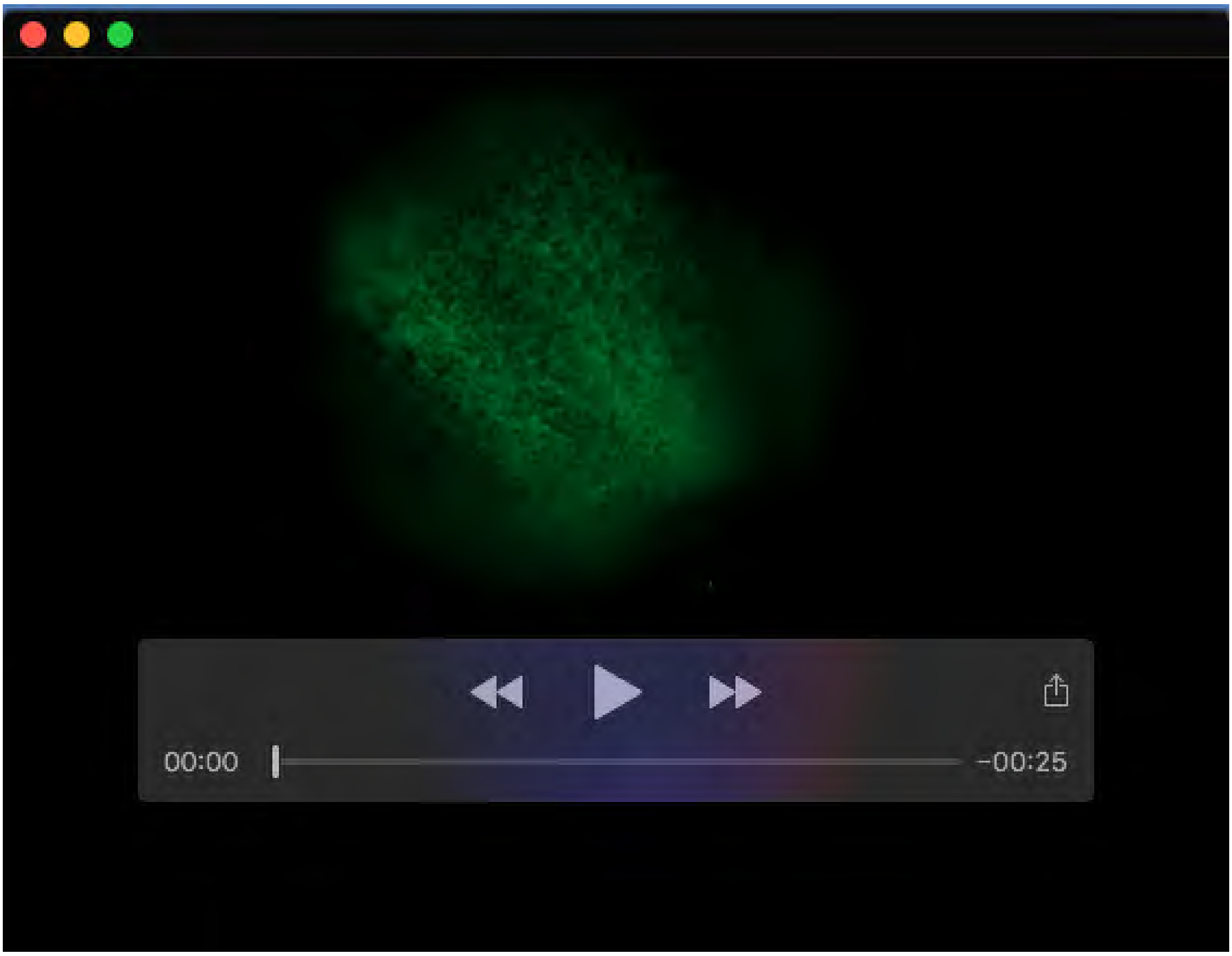

Movie 1. Propagation of PCP in the posterior-to-anterior direction in Xenopus neural plate.

Time-lapse imaging of polarized GFP-Pk3 aggregate formation during neurulation. *Xenopus* embryos expressing HA-Vangl2 and GFP-Pk3 RNAs. Dorsal view is shown. Note progressive posterior-to-anterior formation of GFP-Pk3 crescents. The movie is representative of three independent experiments.

Biology Open • Supplementary information
